# Experimental evidence for snails dispersing tardigrades based on *Milnesium inceptum* and *Cepaea nemoralis* species

**DOI:** 10.1038/s41598-022-08265-2

**Published:** 2022-04-14

**Authors:** Zofia Książkiewicz, Milena Roszkowska

**Affiliations:** 1 Department of General Zoology, Faculty of Biology, Adam Mickiewicz University, Poznań, Uniwersytetu Poznańskiego 6, 61–614 Poznan, Poland; 2 Department of Animal Taxonomy and Ecology, Faculty of Biology, Adam Mickiewicz University, Poznań, Uniwersytetu Poznańskiego 6, 61-614 Poznan, Poland; 3 Department of Bioenergetics, Faculty of Biology, Adam Mickiewicz University, Poznań, Uniwersytetu Poznańskiego 6, 61-614 Poznan, Poland

**Keywords:** Ecology, Zoology

## Abstract

Dispersal abilities in animals contribute to their local genetic variability and species persistence. However, the mechanisms facilitating a short-distance migration of small organisms remain underexplored. In this study we experimentally tested the role of land snails for a fine-scale transmission of tardigrades. We also check the ecological relationship between these two groups, by testing the impact of snail's mucus on tardigrades in anhydrobiosis. All the experiments were conducted under laboratory conditions. As model organisms, we used a tardigrade species *Milnesium inceptum* and a snail species *Cepaea nemoralis.* The selection of the experimental animals was dictated by their co-occurrence in natural habitats and similar atmospheric conditions required for them to remain active. Results of our experiments support the assumption that snails may transfer active tardigrades for short distances. On the other hand, the effect of the snails mucus on tardigrade recovery to active life after anhydrobiosis was negative. Death rates of tardigrades in anhydrobiosis (tun) were higher when affected by mucus compared to mucus-free tuns.

## Introduction

Dispersal is defined as any movement of individuals or propagules with potential consequences for gene flow across space^1^. It affects the distribution of genetic diversity through space by increasing the proportion of total diversity contained within rather than between populations^2^. Therefore, in the context of the current global changes such as habitat fragmentation or climate breakdown, dispersal plays a critical role for species persistence^1^. The dispersal of large animals such as mammals or birds is easy to imagine: a horse can run fast and far thanks to its strong legs while a common swift may fly efficiently thanks to its strong wings and light skeleton. However, our imagination reaches its limits facing the fact that the microorganisms are extremely widespread compared to larger organisms^3^. Scientists paid much attention towards mechanisms that facilitate the spread in small, microscopic animals (e.g.^3,4^). One of the most attention-focusing phenomenon is phoresy. Phoresy is an interaction in which a phoretic animal (termed also as a phoront) latches itself onto a host animal for the purpose of dispersal. Typically phoront is an animal with limited active dispersal abilities that uses a highly mobile host for transportation out of the natal habitat^5,6^. Species dispersing phoretically have been observed in at least 13 animal phyla, 25 classes and 60 orders^7^. The majority of known phoronts are arthropods (Phylum Euarthropoda) in terrestrial habitats^7^. Most records of the phoresy concern mites that were found traveling on beetles (e.g.^8^), bumblebees (e.g.^9^) or centipedes (e.g.^10^). Contrastingly, little is known about the dispersal modes of other tiny and widely distributed invertebrates—tardigrades, commonly known as water bears. The phylum Tardigrada consists of over 1300 species^11–13^ that inhabit freshwater, marine and terrestrial environments throughout the world^14^. Although tardigrades are known from all continents^15–18^, our knowledge is still insufficient to explain all mechanisms responsible for their distribution.

The most famous tardigrades’ “superpower”, that facilitates near and distant colonization of habitats exposed to shorter and longer periods of drying, is their ability to enter anhydrobiosis^19^. Anhydrobiosis can be defined as a tolerance to almost complete dehydration related to near complete loss of body water. Entering anhydrobiosis, the desiccated tardigrade remains in so-called “tun” stage and may stay in this state for several years and resume active life when water becomes available again^20^ (the longest documented survival time in anhydrobiosis for heterotardigrade is 20 years^21^ and 15 years for eutardigrade^22^). Persisting in anhydrobiosis is a high-cost process, because the longer the organism is dehydrated, the longer it will take to return to active life^20^. On the other hand, the animals in the tun stage may be easily spread by wind or may be taken by birds for a long-distance journey^23^. The mechanisms responsible for a fine-scale dispersion of tardigrades remained however poorly explored. Facing the climate crisis and biodiversity loss, studies on short-distance dispersion are urgently needed since they contribute toward a better understanding of the local transfer of alleles^24^. Such gene flow may have in turn a decisive impact on local adaptations and therefore also population survival^24^.

Tardigrades live in specific microhabitats and always require at least a film of water surrounding their bodies to become active in terrestrial environments^20^. The conditions favouring tardigrades’ activity may occur even only for a few hours a year^25^. This way water bears walk rather short distances on their own and their movements are limited to very nearby spaces^25^. Instead, they break microhabitats’ barriers by moving passively, using the force of wind or water rivulets^3^. Although the role of abiotic factors for water bear dispersion was documented, the role of animal-mediated short-distance transportation remains almost unexplored. When looking for a perfect mobile candidate for tardigrades transportation, it would be ideal if the host animals have moist integuments as tardigrades need high humidity to stay active. Another condition is co-occurrence in the same habitat, and the last one—activity in a convergent conditions and time. Following these assumptions, land snails seem to be perfect vehicles for tardigrades. Some gastropods and tardigrades may co-occur in the same habitats (e.g. concrete walls, forest litter)^14,26,27^. Additionally, snails (similar to tardigrades) become active when humidity elevates and have permanently moist skin constantly moisturized by mucus. Although land snails are known for their low motility, they move faster and over much larger distances than tardigrades^25,28^. Shcherbakov et al.^25^ demonstrated that tardigrades move less than 23 mm/h while land snail belonging to a species *Cepaea nemoralis* may disperse at a rate of 25 m^28^. Moreover, snails may easily walk through microhabitats impenetrable for tardigrades, passing through anthropogenic barriers such as small roads or lawns. This way, snail-driven dispersal has potentially significant advantages for genetic diversity of tardigrades metapopulations.

The relationship between tardigrades and land snails is poorly recognized, despite the potential phoront-host match. To date, only three publications involving terrestrial gastropods and water bears were released^27,29,31^. All these papers concerned relations between snails’ digestive track and tardigrades. The oldest of the above-mentioned study authored by Fox^29^ describes alive tardigrades from the feces of a common snail *Bulimulus exilis*^30^ in the city of San Juan, Puerto Rico. The author also collected sand and plant samples from the environment where the snails were found and underlined that no tardigrades have been found in the samples. Prior to the examination, all snails were washed and transferred to clean Petri dishes to eliminate the possibility of transferring tardigrades on the snail’s body. Tardigrades were present only in snail’s excrement. The presence of tardigrades in the snail feces and their absence in the habitat from which the snails were collected suggested that the tardigrades could be transferred within the snail to places more or less distanced from their initial site. A year later, Fox and Garcia-Moll^31^ released a publication with a description of the new tardigrade species *Claxtonia molluscorum*^31^ found in *B. exilis* feces. The third snail-tardigrade publication was also authored by Fox^27^, where he explains possible ways tardigrades got into the gastrointestinal tract of snails to be finally found in feces. Fox^27^ found that *C. molluscorum* may co-occur with *B. exilis* on cement walls. Tardigrades dwelled within the dark molds while snails actively crawled on the same wall. While foraging, snails possibly scratched out the tardigrades with the radula and swallowed them up. After Fox’s paper in 1966, for over half a century, studies on the relationships between tardigrades and molluscs were abandoned.

In our research we went back to the study on interactions between snails and tardigrades. We tested three following hypotheses: (1) snails have a significant effect on dispersion of active tardigrades, (2) dispersal efficiency depends on the substrate, and (3) snails’ mucus interact with tardigrade tuns affecting their recovery from anhydrobiosis. We focused on the species that co-occur in the natural environment: *Milnesium inceptum* (representative of the phylum Tardigrada) and *Cepaea nemoralis* (phylum Mollusca) and arranged their meeting.

## Material and methods

### Species used in the experiments

**Figure 1:**
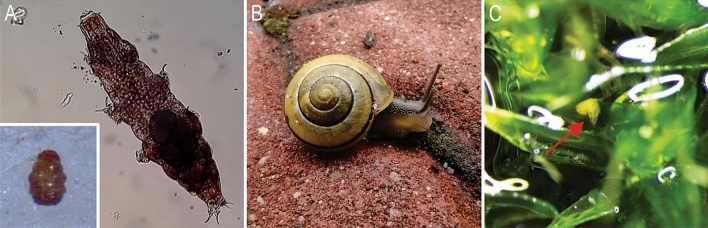
Model animals used in experiments: (**A**) *Milnesium inceptum*; insert shows tardigrade in the tun state; (**B**) *Cepaea nemoralis* in its natural environment; (**C**) a tardigrade that appeared on moss surface during in vivo observation of rehydrated moss cushion (red arrow). Figures were assembled in Corel Photo-Paint 2017 (http://www.corel.com).

*Milnesium inceptum*^32^ (Fig. 1A, a picture taken using Olympus BX41 Phase Contrast light Microscope associated with Olympus SC50 digital camera) is an obligatory predatory species with the body length ranging from 326 to 848 μm. It feeds on rotifers, nematodes and other tardigrades and lays smooth eggs in exuviae. To stay active, *M. inceptum* needs a thin water film around its body^14^. The species inhabits places exposed to shorter and longer periods of drying i.e. frequently drying mosses growing on cement walls^32^. Till now it was reported in Poland, Germany, Japan, Switzerland and Bulgaria^32^. At the same time, it is a perfect organism for our research because (1) it is large and easy to observe, (2) it tolerates frequent periods of entering and leaving anhydrobiosis, (3) it easily creates a tun stage. *Milnesium inceptum* for experimental purposes were acquired from a moss sample from a cement wall in Poznań, Poland (52°24′15″N, 16°53′18″E). The extraction of tardigrades was conducted under stereomicroscope (Olympus SZ51) using standard methods^33^. Then specimens, further used in our experiments, have been cultured based on protocol proposed by Roszkowska et al.^34^. Only fully active, adult specimens were selected for the experiments.

*Cepaea nemoralis*^35^ (Fig. 1B, a picture taken using Motorola g(9), Camera version 7.3.63.53-whitney) is a stylommatophoran European land snail species, which is widespread and common throughout the continent^36^. The average maximum shell diameter is 20 to 22 mm^37^. It feeds on plant materials available, yet has a strong preference for dead and senescent herbs^38^. *C. nemoralis* occurs in variable habitats (frequently in synanthropic ones) such as forests, meadows, gardens, near shrubs or dunes^36^.The period of its activity falls on the growing season; it usually comes out of the shell and crawls when the air humidity reaches 70% or more, independently from solar radiation and air temperature^28^. The species is a good model for our study due to its: (1) large size compared to tardigrades, and (2) co-occurrence with *M. inceptum* in natural environments. Individuals of *C. nemoralis* were harvested from anthropogenic environment: gardens adjacent to detached houses (52°25′28″N, 16°46′52″E). Snails were collected from plants, cement walls and ground surfaces. After collection, all *C. nemoralis* specimens were washed-up and placed in 30 L (480 × 360 × 252 mm) transparent plastic box with mesh covering for ventilation. Soil and rocks were placed in the box allowing to maintain a moist shelter for snails, and a sepia was used as a source of a calcium. Animals were fed with lettuce, cabbage and nettle twice a week and sprinkled with water to stimulate their activity. Box containing snails was kept in a rearing room, at 17 °C in 12:12 photoperiod. Snails were kept in the box for 1.5 months prior to the experiments. For the experiments we used only adult animals. The snails were checked under Olympus SZX7 stereomicroscope prior to the experiment to ensure they were free of tardigrades.

### Pilot studies

#### Does the tardigrades’ distribution within a moss cushion enable tardigrade-snail contact?

To check whether tardigrades may come into a close encounter with the snail in the natural environment (which would be impossible if the tardigrades were only present in the lower layers of the moss), we investigated the distribution of water bears within moss cushions. The observations were performed for 6 samples of dried moss cushions (ca. 1 cm high and 3 cm in diameter). The moss containing *M. inceptum* specimens, was collected from a concrete wall in Poznań, Poland (52°24′15″N, 16°53′18″E), the same from which tardigrades were initially collected for the culturing purposes. Three moss cushions were rehydrated, and left for 3 h followed by further observation to check whether tardigrades may actively move across the moss cushion. On the remaining three moss samples, a horizontal cut was made through the center of the moss cushion to check in which layer tardigrade tuns are present while the moss remains dry. The extraction of tardigrades from separated layers was conducted under stereomicroscope (Olympus SZ51) using standard methods^33^.

Within the dry moss cushions tardigrades were present in both the upper and lower moss layers. We did not observe any difference in the number of individuals of *M. inceptum* that would be dependent on the moss layer. A total of 353 tardigrades were extracted from one moss cushion (dry weight of moss = 0.332 g), what gives the density of tardigrades per 1 g of dry moss sample equal to 1063 specimens. The observation of rehydrated moss cushions conducted in vivo using Olympus SZX16 stereomicroscope associated with Olympus DP74 digital camera and cellSens software revealed that single active tardigrades may also appear on the moss surface (Fig. 1C, red arrow). Therefore, observed in the pilot studies tardigrades distribution within the moss cushion enables tardigrade-snail contact.

#### Is it possible for a tardigrade to take a snail ride?

The initial observations were carried out for snails and tardigrades to check whenever a tardigrade may be transferred by a snail. In total, 10 snails and 20 active tardigrades were used. Two variants of Petri dishes (ø 90 mm) were prepared: (1) with smooth and (2) scratched bottom, to avoid and allow tardigrade attachment to the bottom of the dish, respectively. We repeated the observation five times per option. For each single observation we used one snail and two tardigrades.

Snails and tardigrades were split equally between the pilot’s experimental options (in total 5 snails and 10 tardigrades per option). We checked whether tardigrades may be transferred by snails by putting tardigrades in the drop of water in the center of a Petri dish and releasing an active snail to crawl through the drop. In total, in the case of the smooth-bottom option, three tardigrades glued to the snail’s body within which two were moved to a distance up to a few centimeters. The third one fixed to a snail’s leg and had a potential to be transferred to a greater distance. In the case of the dishes with the scratched bottom, we did not notice any transfer. Tardigrades were attached tightly to the dishes’ bottom and remained unmoved after the snail had passed through them. Therefore, the observation in the pilot study confirmed that tardigrades may stick to snails’ body and be transferred by a gastropod at least when the substratum (bottom of the dish) is smooth.

### Experimental design

#### *Experiment 1*. Do snails have a significant effect on tardigrade dispersion that depends on the substrate type?

As the laboratory environment offers limited possibilities to reflect natural conditions, we aimed to create an environment similar to the natural one by eliminating as many artificial elements as possible and, at the same time, enabling observation and data collection. To imitate a natural microhabitat of water bears we used a piece of moss as a substrate. Moss is a natural shelter and a hunting space for these animals, and a gripping surface that prevents them from being easily carried away by a stream of water or wind. The moss *Vesicularia dubyana*^39^ used in the experiment was purchased in an aquarium shop and was derived from an in vitro culture. It was checked under Olympus SZX7 stereomicroscope prior to the experiment to ensure it was free of tardigrades. For experimental purposes we used plastic ventilated boxes with dimensions 950 mm × 950 mm × 600 mm, tightly closed with a plastic lid. The bottom of each box was scratched with sandpaper in order to (1) imitate a rough surface of a concrete wall to which mosses are attached in the natural environment; (2) allow tardigrade locomotion. At the same time, moss and (unfortunately) plastic elements are quite common surroundings of *C. nemoralis* frequently found in anthropogenic habitats^36^.

Using transparent, non-toxic aquarium silicone, a square with a side length of 3 cm and a height of 0.5 cm was mounted on the bottom of the box. Before starting the experiment, the tightness of the square silicone barrier was checked by pouring 2.5 ml of water inside and leaving the boxes for observation for 24 h. After this time, all silicone squares turned out to be impermeable to water.

**Figure 2:**
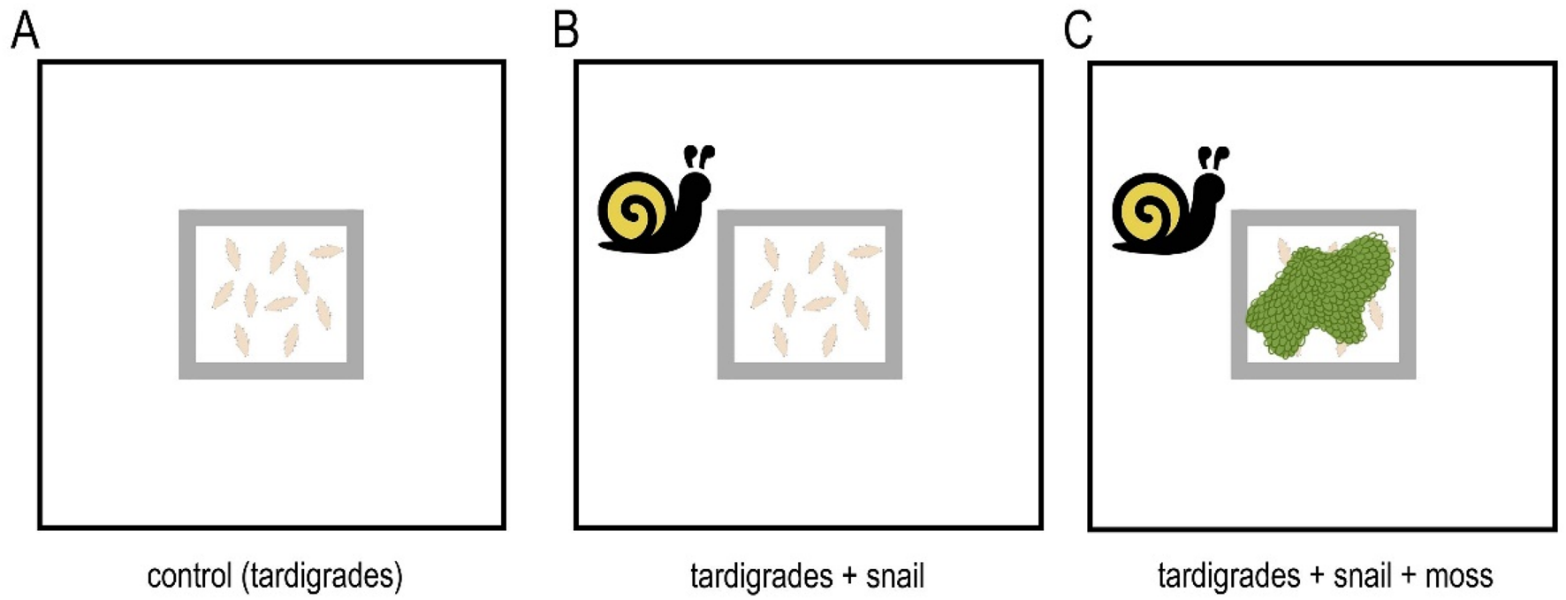
Graphical representation of three designed experimental options of the experiment 1. (**A**) 10 tardigrades in the silicone square (control (C)); (**B**) 10 tardigrades in the silicone square and one snail placed in the box (tardigrades + snail (TS)); (**C**) 10 tardigrades in the silicone square, one snail placed in the box and additional piece of the moss added as a barrier between tardigrades and snail (tardigrades + snail + moss (TSM)). Figures were assembled in Corel Photo-Paint 2017 (http://www.corel.com).

Boxes for each of the experimental option, namely: (A) control (further in the text referred as C), (B) tardigrades + snail (referred as TS), and (C) tardigrades + snail + moss (referred as TSM, see Fig. 2), were prepared in a following way: 2.5 ml of water was added to the scratched bottom of the box inside the silicone square and 7.5 ml to the area outside of the silicone square to enable survival and active locomotion of tardigrades on both sides of the silicone barrier. Then, 10 active individuals of *M. inceptum* taken from the culture were transferred to the center of the silicone square. It was repeated for 90 boxes (30 boxes per each C, TS and TSM option). Therefore we used 300 tardigrades per each experimental option which gives 900 tardigrades in total for all experimental options. In case of 30 boxes with TSM option, a piece of moss (ca. 2.5 cm in diameter) was added. It was situated in the center of the silicone square, just after the tardigrades were placed at the boxes in order to isolate tardigrades from the snail during the experiment.

Finally, in the boxes targeted for TS and TSM experimental options, one adult and active individual of *C. nemoralis* snail was placed in each box outside the silicone square. In total, 60 snails were used (30 individuals per experimental option).

The boxes were then placed in the rearing room (17 °C, 80% of humidity, photoperiod 12:12) for 72 h. After this time, the number of tardigrades inside and outside the silicone square was counted (both: live and dead) separately for each box, using Olympus SZX7 stereomicroscope.

#### Experiment 2. Effect of the snail’s mucus on tardigrade recovery to active life after anhydrobiosis

##### *Milnesium inceptum* anhydrobiosis protocol

Only fully active, adult specimens of medium body length were selected for the experiment. The animals were transferred to ø 3.5 cm vented Petri-dishes with bottom scratched by sandpaper to allow tardigrade locomotion. Five tardigrade individuals were placed to each Petri dish together with 450 µl of water and then dehydrated. In total, 16 Petri dishes with 5 tardigrades on each were prepared. Dehydration process lasted 72 h and was performed in the Q-Cell incubator (40–50% RH, 20 °C, darkness). After that time tardigrade tuns were kept under the abovementioned conditions for 7 days.

##### Impact of the snail’s mucus on tardigrade tuns

**Figure 3:**
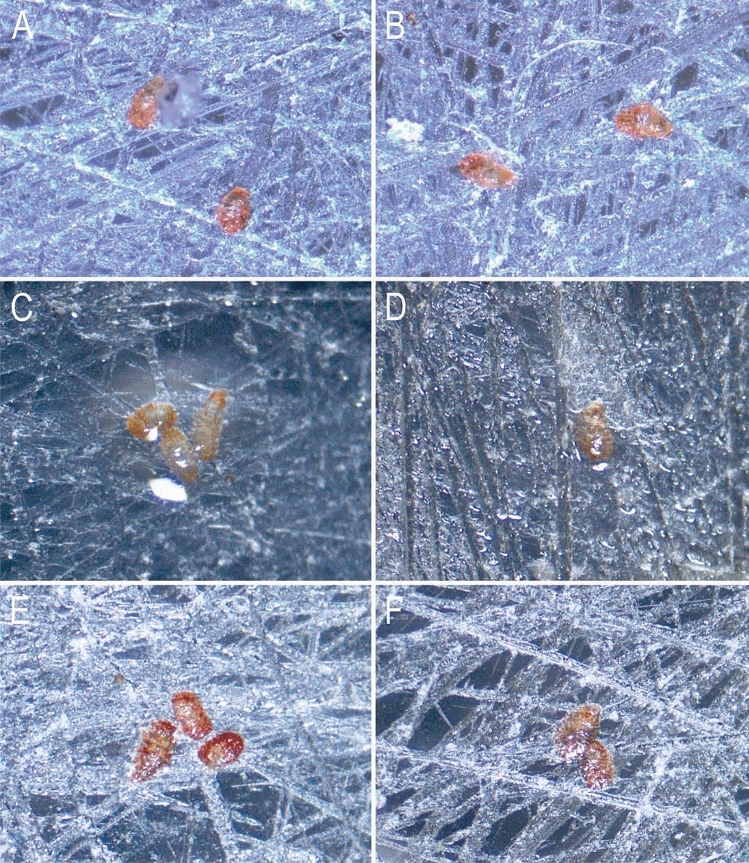
*Milnesium inceptum* tuns: (**A**,**B**) before contact with snail mucus; (**C**,**D**) coated with wet snail mucus; (**E**,**F**) coated with dry snail mucus. Figures were assembled in Corel Photo-Paint 2017 (http://www.corel.com).

After 7 days of anhydrobiosis, one individual of *C. nemoralis* was transferred to each dish with tardigrade tuns and was left there for 1 min allowing the snail to actively crawl over the tuns. 30 min after the snail was removed from the dish, tardigrade tuns were observed under the Olympus SZX7 stereomicroscope for any animal movements. Then, all covered and vented dishes were left in the Q-Cell incubator overnight. After 24 h, the dried tuns were rehydrated by adding 3 ml of water to each Petri dish to check whether snail’s mucus affected mortality rates of tardigrades. After 3 and 24 h following rehydration tardigrade tuns were observed for any animal movements. Pictures of tuns were taken using Olympus SZ61 stereomicroscope associated with Olympus UC30 camera (Fig. 3). As reference data on the rehydration of the *M. inceptum* tuns free of the snail’s mucus, we used the data from Roszkowska et al.^20^ who tested anhydrobiosis survivability of above-mentioned species. Individuals used for the tuns preparation in the control option were collected from the same laboratory breeding stock, and prepared at the same laboratory conditions as those used in our experiments^20^.

### Statistical analyses

The number of tardigrades relocated in each experimental option (C, TS and TSM) was compared with a one-way ANOVA randomized version using RundomPro 3.14 software^40^. We used non-parametric methods because of the lack of normality. Differences were considered significant at p < 0.05.

## Results and discussion

### Effect of the type of a substrate on transporting tardigrades by snails

**Figure 4:**
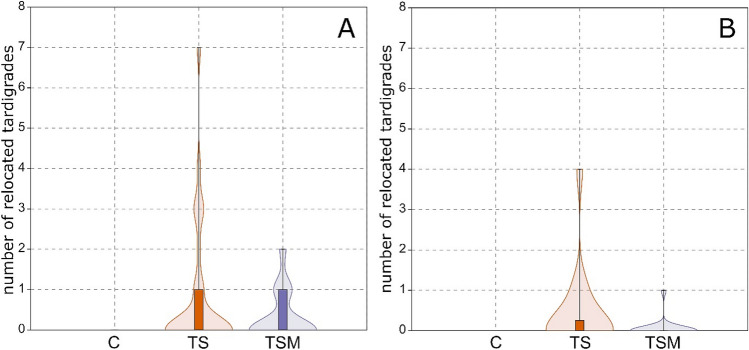
Violin plots with box plots showing number of tardigrades relocated for (**A**) dead and alive tardigrades transferred; (**B**) tardigrades being alive during inspection. The plot depicts data for each experimental option: T—tardigrades, TS—tardigrades with snails, TSM—tardigrades, snails and moss. Whiskers show maximum and minimum values of tardigrades relocated per sample at each option. Graphs were made with PAST 4.0 software (http://www.nhm.uio.no/english/research/infrastructure/past/).

Up to now almost nothing is known about transferring active tardigrades (putative phoresy) by other animals in terrestrial habitats. Two reports are known on tardigrades that were transported in digestive system of animals: (1) tardigrades found in feces of *B. exilis* snail (mentioned in the “Introduction” section)^29^ and tardigrade tuns found in the feces of birds belonging to the *Attagis malouinus* species^41^. In both cases, the animals entered the gastrointestinal tract most likely accidentally with food. However, tardigrades survived, being transported long distances, where they could settle into new habitats. It was also proved that birds can transfer tardigrades in their feathers as well as collect them together with nesting materials^23,42^. Our experiments have shown that snails may be also considered as vehicles for tardigrades. In experimental dishes where snails were absent, tardigrades were not relocated out of the silicone square. Therefore, we assume the snails were responsible for tardigrade transportation. However, it needs to be underlined that the transport efficiency could depend on the substrate type (Table 1). During our experiment, some tardigrades died, however we were unable to determine at which point of the experiment it happened. Since in our initial observation (pilot studies) all tardigrades survived transfer, we suspect that tardigrades could have died as a result of low oxygen level and/or water contamination by snail’s feces. Relatively small dimensions of the box and low water volume, which was additionally crawled through by snails favored contamination. However, such cause of tardigrades death is only our suspicion, therefore we decided to perform analyses for both (1) all alive and dead tardigrades found outside of the central square (transferred by snail) as well as (2) for alive tardigrades only (Fig. 4).

**Table 1:** Total number of tardigrades relocated out of the square and not relocated, alive and died for each experimental option (total number of tardigrades for each experimental option was 300): C—control; TS—snail + tardigrades; TSM—snail + tardigrades + moss.

Experimental options	C	TS	TSM
**Relocated**			
Total number	0	38	12
Mean	0	1.3	0.4
Max	0	8	3
Min	0	0	0
SD	0	7.0	2.3
Alive	0	13	2
Mean	0	0.44	0.07
Max	0	4	1
Min	0	0	0
SD	0	1.1	0.3
Dead	0	25	10
Mean	0	0.84	0.34
Max	0	7	2
Min	0	0	0
SD	0	1.7	0.6
**Not relocated**			
Total number	300	262	288
Alive	235	152	107
Dead	65	110	181

Standard deviation (SD) and mean (Mean) given for relocated tardigrades as well as minimum (Min), and maximum (Max) number of transferred tardigrades per square.

The one-way randomized ANOVA showed that both models are statistically significant (the model for alive and dead tardigrades: F = 5.913, P = 0.002; the model for alive tardigrades only: F = 3.154, P = 0.036). However, considering the results of the post-hoc analyses for alive and dead tardigrades, it seems that snails affect significantly tardigrade transfer independently from the substratum (C vs TS and C vs TSM; Table 2, Fig. 4).

**Table 2:** Results of post-hoc tests (two sample rand. Tests + Holm's correction; # of rand. = 10,000) for one-way ANOVA randomized run for transferred alive and dead tardigrades (F = 5.913, P = 0.002), as well as alive tardigrades only (F = 3.154, P = 0.0356).

	Alive and dead tardigrades	Alive tardigrades
	p-value (significance)	
C vs TS	0.003	0.036
C vs TSM	0.010	> 0.05
TS vs TSM	> 0.05	> 0.05

*C* control, *TS* snails and tardigrades, *TSM* snails, tardigrades and moss.

On the other hand, when considering the analyses for alive tardigrades only, the result was significant between C (control) and TS (tardigrades + snail) options only. Since no statistical significance was shown between C and TSM options, it seems that the moss could have impeded the transfer of alive tardigrades (however did not stop the transfer completely, see Fig. 4). Our pilot study revealed that tardigrades are distributed more or less evenly throughout the dry moss cushions. When the moss was rehydrated only some single active individuals appeared on the cushion’s surface. Therefore the opportunities for a snail to transfer a tardigrade seem to be less frequent compared to the none-moss option when all individuals are exposed to a snail. Our study confirms this assumption. In the TS option, tardigrades were transferred by snails in larger numbers (Fig. 4). It should be however underlined that the transport of alive tardigrades by a snail (even when no moss is available) may be hindered as tardigrades are usually tightly fixed to the substratum by their claws. That speculation can be supported by our pilot study. The scratched substratum enabled tardigrades to attach tightly to the bottom of the experimental dish. The adhesion was strong enough to prevent the tardigrade transfer by a snail (see “Is it possible for a tardigrade to take a snail ride ?” in the “Material and methods” section). On the other hand, dead and detached from the surface, the individual may be transferred by a snail more easily. Further, we suspect that such the snail-mediated transfer may apply even more to tardigrade exuvia with eggs that are not attached to the substrate and can passively float in the water. Also, a similar situation will occur with the freely-laid tardigrade eggs that are laid directly into the environment. Such egg chorion has many processes on its surface which often end with various types of filaments, spikes or discs. These structures seem to be a perfect tool for anchoring to various structures facilitating transfer and/or settlement in new areas. However, hypotheses testing the eggs transfer as well as the role of the chorion appendages in settlement in new areas still await for experimental verification.

The short- distance dispersion (including animal-mediated transportation) may have an advantage over the dispersion driven by untargeted natural forces, wind especially^43^. Although a strong air gust may probably take tardigrades more often than snails could do, and may carry them over many kilometers (Zawierucha et al.^44^ studying tardigrade habitats on glaciers showed that the wind may transfer glacier tardigrades even to other glaciers more than 1000 km away), the environment at the final stop is random and may be hostile for a tardigrade. In contrast, a snail is a vehicle orientated to a particular type of a microhabitat. That is why we decided to consider the role of land snails in dispersing tardigrades over short distances. We suspect that if a snail will take a tardigrade (or tardigrade egg) for a ride, the passenger will be dropped off within a suitable environment and due to low active dispersal abilities of tardigrades, transfer of an individual to even several dozen centimeters may be beneficial for local genetic variability (and this way—metapopulations). Although we provided experimental evidence that tardigrades may be transferred by land snails, the intensity of this process remains unknown. It should also be remembered that a quantification of the role of any potential dispersal on the biogeography of tardigrades and settlement of new habitats is unavailable, and the role of animal- (including other invertebrate species), wind- and water- mediated effects on dispersal is still discussed^3^. Till now, no quantification of tardigrade dispersal is possible, therefore we can only hypothetically discuss which dispersion factor is the most effective for tardigrades.

### Effect of the snail’s mucus on tardigrade recovery to active life after anhydrobiosis

**Figure 5:**
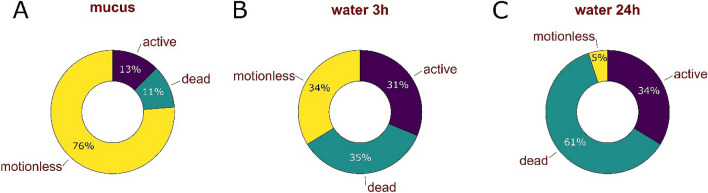
Percentage of active, dead and motionless tardigrade individuals: (**A**) 30 min after contact with snail mucus, (**B**) tuns covered with dried mucus 3 h following rehydration, (**C**) tuns covered with dried mucus 24 h following rehydration. Graphs were made with PAST 4.0 software (http://www.nhm.uio.no/english/research/infrastructure/past/).

The locomotion mucus of land snails consists mainly of water (up to 98%) as well as large, carbohydrate-rich molecules with some relatively small proteins^45,46^. Terrestrial snails usually need a less humid environment than tardigrades to remain active, therefore we suspect that in a natural environment it is highly possible that snails may crawl over mosses and, at the same time, over tardigrade tuns. The experiment verifying whether the snail mucus affects tardigrade recovery to active life after anhydrobiosis showed that tardigrades can survive after the mucus contact. Tardigrades, immediately after contact with the mucus, start to rehydrate, as shown in the attached photographic documentation (Fig. 3). However, their later recovery to full activity was much longer in the case of the mucus-glued tuns than in the control (according to Roszkowska et al.^20^) 98% rehydrated tuns of *M. inceptum* recovered to full activity after spending 7 days in anhydrobiosis). In three individuals we also observed a problem with releasing from the mucus envelope, despite the return of tardigrades to full activity (observation 24 h after rehydration). Only after a mechanical detachment (with a pointed spatula, without damaging the tardigrade) the animals began to move along the bottom of the dish. We also noticed that the mortality rates of tardigrade tuns after the contact with the snail mucus were higher than in the control where 98% of tardigrades survived and become active after 24 h following rehydration (34% in case of tuns covered by mucus) (Fig. 5). We suspect that the mucus dried up too quickly and the tardigrades undergoing rehydration were unable to react quickly enough to the drying microenvironment. As a result, it was difficult to form correct tuns again in such a short time. The very quick disappearance of access to water could have contributed to the insufficient performance of the cytoprotective strategies applied by tardigrades while forming proper tuns (including synthesis and accumulation of molecules that enable the prevention of damage to membrane and macromolecule functions by stabilizing their structures). This would explain the higher mortality of animals after 3 and 24 h following the final rehydration of tuns covered with dry mucus (Fig. 5). We also suspect that entering anhydrobiosis following such a short period after rehydration could have been also a tremendous effort for the animal which additionally increased mortality rates of tardigrades.

It is also worth mentioning that the snail mucus has a proven antibacterial effect, showing an inhibitory effect against strains of gram-positive and gram-negative bacteria^47^. In recent years, some studies on the composition of the microbiome in tardigrades has been started. It is supposed that the anhydrobiotic capability of tardigrades could influence their microbial community, maybe even constraining bacterial species that could develop a stable association with tardigrades^48^. On the other hand, the role of the microbiome composition in the process of tardigrade successful anhydrobiosis remains unknown. If such a correlation was proven, there would be a possibility that the antibacterial activity of the snail mucus could somehow influence the tardigrade microbiome responsible for the effective entry into anhydrobiosis.

## Conclusions

Our experiments showed that the snail-tardigrade interaction may have both positive and negative effects on tardigrades. We proved that snails may be considered as vectors for a fine-scale transfer of tardigrades. On the other hand, our experiments also showed that land snails’ mucus may have a damaging effect on tardigrade tuns because, according to our results, mucus-glued dehydrated tardigrades showed higher mortality rates. We speculate that land snails may be considered as one of the short-distance dispersion factors for tardigrades, potentially affecting genetic structure of tardigrade’s local populations. However, in the current state of knowledge, its effectiveness remains difficult to quantify.
